# Full three-dimensional Poynting vector flow analysis of great field-intensity enhancement in specifically sized spherical-particles

**DOI:** 10.1038/s41598-019-56761-9

**Published:** 2019-12-27

**Authors:** Liyang Yue, Bing Yan, James N. Monks, Rakesh Dhama, Chunlei Jiang, Oleg V. Minin, Igor V. Minin, Zengbo Wang

**Affiliations:** 1 School of Computer Science and Electronic Engineering, Bangor University, Dean Street, Bangor, Gwynedd LL57 1UT UK; 2 College of Electrical and Information Engineering, Northeast Petroleum University, Daqing, 163318 China; 3 National Research Tomsk Polytechnic University, Lenin Ave., 30, Tomsk, 634050 Russia; 4 National Research Tomsk State University, Lenin Ave., 36, Tomsk, 634050 Russia

**Keywords:** Micro-optics, Micro-optics

## Abstract

The Poynting vector plays a key role in electrodynamics as it is directly related to the power and the momentum carried by an electromagnetic wave. Based on the Lorenz-Mie theory, we report on the focusing effect of a spherical particle-lens by properly analysing the Poynting vector maps. Conventional two-dimensional (2D) maps showing Poynting vector magnitude and direction in a given plane cannot deliver information on three-dimensional (3D) directivity and vectorisation in key regions of singularities, such as vortexes and saddle points, due to poor expressiveness. In this article, an analytical 3D mapping technology is utilised to track the field-features passing through the singularities of the distribution of the Poynting vector in a spherically dielectric mesoscale particle-lens. We discovered that the spheres with the certain size parameters can stimulate extremely large field-intensity at singularities and then form two circular hotspots around the sphere poles. An astonishing large ‘heart-shape’ 3D Poynting vector circulation, which cannot be predicted by conventional 2D mapping analysis, is found to provide a great angular variation within an enormous range in these spheres. We anticipate that this effect will contribute to the field-enhancement phenomena, such as surface enhances Raman scattering, surface enhances absorption, super-resolution imaging and others.

## Introduction

The energy stored in the electric and magnetic fields is transmitted at a certain energy flow rate which can be calculated based on the Poynting theorem derived back in 1884^1^. To represent the directional energy flux of an electromagnetic field^2,3^, it is acceptable to use the Poynting vector which is given by a cross product of the electric and magnetic fields (measured in V/m and A/m, respectively). Therefore, Poynting vector is a dimensional vector quantity and it is expressed as VA/m^2^ or W/m^2^, because electric and magnetic fields are both vectors. Computer-aided 2D mapping is a common plot method to characterise the Poynting vector in a certain electromagnetic field. Contour, colour, and field-line with arrows are normally used to denote magnitude and direction of the Poynting vector. However, in fact, a 2D mapping cannot accurately reflect the behaviour of Poynting vector at singularities because the conventional representations of Poynting vector are misleading over there. The singularities in a 2D plot representation of the Poynting vector, such as saddle point or vortex, may indicate a complex coupling behaviour. This behaviour is the result of the Poynting vector flow through singularities along the spiral trajectories^4,5^. In this case, a 2D colour map can only determine positions of the singularities of Poynting vector, but destinations of the field-lines through these points are still unknown. The corresponding limitation of 2D colour mapping in Poynting vector representation was also found by the other researchers. Soukoulis *et al*. pointed out that 2D Poynting vector plot cannot perfectly reflect the real situation in the experiment of negative refraction for a 3D photonic metamaterial due to a poor interpretation of multiple singularities in it^6^. The similar circumstances of multiple Poynting vector singularities have been repeatedly discovered in laser devices, metamaterials, and super-resolution lenses^7–9^, which indicates that there is a strong demand to develop a spatial 3D tracking algorithm for the Poynting vector plot in photonics research.

Lorenz-Mie theory fully describes the optical absorption and scattering of light by a homogeneous sphere, which verifies that a jet-like near-field focus situates near the shadow surface of the sphere in the certain range of *a/λ* (*a*, particle radius, and *λ*, light wavelength)^10^. Such kind of focusing effect is named as ‘photonic jet’ and has been attracted much attention since 2000^11–13^. It is known that photonic jet can deliver a strong field-intensity enhancement with a long-distance propagation (for several wavelengths) and possibly constitute a near-field super-resolution optical system^13^. Many approaches to sphere transformation, e.g. metalens, assembly of nano-fibres, and addition of a pupil-mask, were carried out to further reduce the transverse dimension and enhance field-intensity for photonic jet^14–17^. It is widely accepted that singularities of Poynting vector should be related to the above approaches, nevertheless, there is no mature mapping technology that can perfectly visualise the corresponding dependence due to the meaningless interpretation of Poynting vector presented as a 3D map. For that, Wang *et al*. systematically characterised features of Poynting vector in a small particle and its focus and claimed that the higher orders of optical resonance should be taken into account^18^. Fu *et al*. and Mundy *et al*. formulated the analytic expressions for single-scattering properties of a spherical sphere in an absorbing medium using a Poynting vector plot. However, these expressions were failing to graphically quantify the influence of singularities at the specific locations^19,20^. So far, there is no established theory to relate the elements of particle size, field-intensity enhancement of focus, and Poynting vector distribution. As an isolated physical phenomenon, giant field-intensity enhancement in spheres with particular sizes, was occasionally observed in the form of explosion of few spheres in the initial research of laser cleaning of micro/nano-particles with random sizes. However, there was no comprehensive model that could explain this phenomenon back then^21,22^.

Teflon is a widely-used material for terahertz (THz) sensing and imaging nowadays due to its outstanding low-loss properties and low cost^23,24^. Compared with super-resolution lenses made of lossy metals, e.g. gold and silver, a Teflon super-resolution lens or a similar dielectric super-resolution lens is not limited by the intrinsic loss relying on an extremely small extinction coefficient, *k*, (nearly 0) of the material in the THz band. Minin *et al*. generated a photonic jet (terajet) in the THz band by using a dielectric non-resonant Teflon cuboid particle^25–30^. They showed the super resolution effect by using lens made of Teflon and PMMA spherical particles^31^. However, these studies did not involve any investigation about the size screening for particles. In this paper, we use for the first time a 3D mapping technology to track field-lines passing thought the singularities and hotspots (critical points) in a Poynting vector distribution map of a spherical Teflon particle-lens (refractive index *n* = 1.43^32^). Maximal electric field-intensity (|*E|*^2^) enhancement was analytically calculated varying the size parameter, *q* between π and 20π. The critical points of Poynting vector found in the sphere were set to the initial positions of 3D field-line to study the giant field-intensity enhancement in spheres.

## Results and Discussions

**Figure 1:**
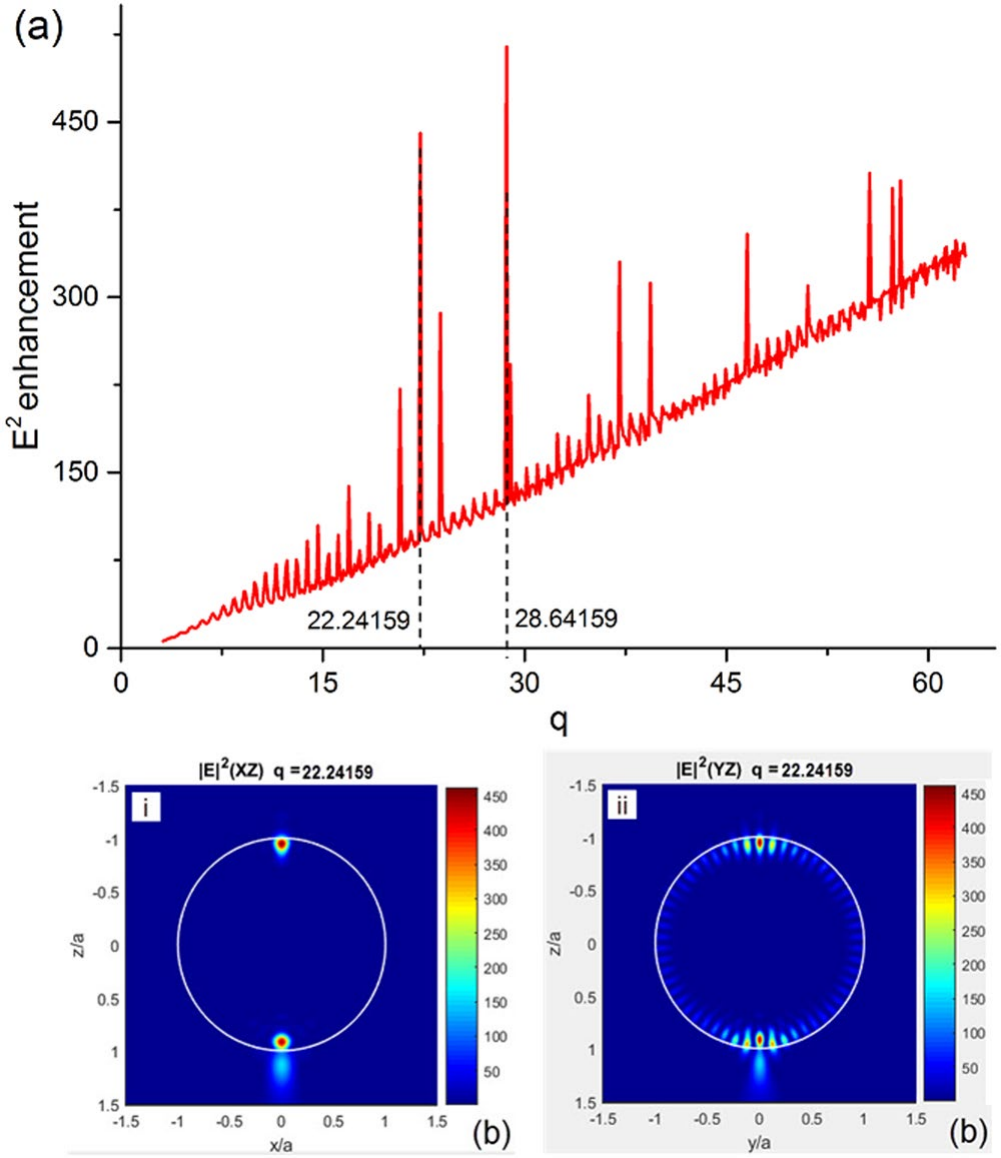
**(a)** Peak |*E|*^2^ field-intensities for the Teflon spheres versus size parameter, *q*. The distributions of |*E|*^2^ field-intensity in the *xz* plane **(b)** i and the *yz* plane **(b)** ii when *q* = 22.24159.

Figure 1(a) summarises peak |*E|*^2^ field-intensities along the central axis as a function of size parameter, *q*, for all Teflon spheres in this study. An increasing tendency is manifested with the regular oscillations for this curve. Two well-defined peaks associated with the giant enhancement reach values of 438 and 514 at *q* = 22.24159 and *q* = 28.64159, respectively. These values are far larger than those for the Teflon spheres with the neighbouring size parameters. We select the sphere of *q* = 22.24159 to plot the distributions of |*E|*^2^ field-intensity and Poynting vector to track the 3D field-lines passing through the critical points to analyse this giant enhancement effect. Figure 1(b)i,ii show the distributions of |*E|*^2^ field-intensity in the *xz* and *yz* planes, respectively. We note that the photonic jet (the light-blue-colour area below the sphere) is not of the largest magnitude. The areas possessing maximum |*E|*^2^ field-intensity symmetrically form two circular hotspots at the sphere poles in the *xz* place, as shown in Fig. 1(b)i, otherwise three smaller hotspots (the central one is larger) can be found in the same regions with the Whispering-Gallery mode effect shown in Fig. 1(b)ii for the *yz* place.

**Figure 2:**
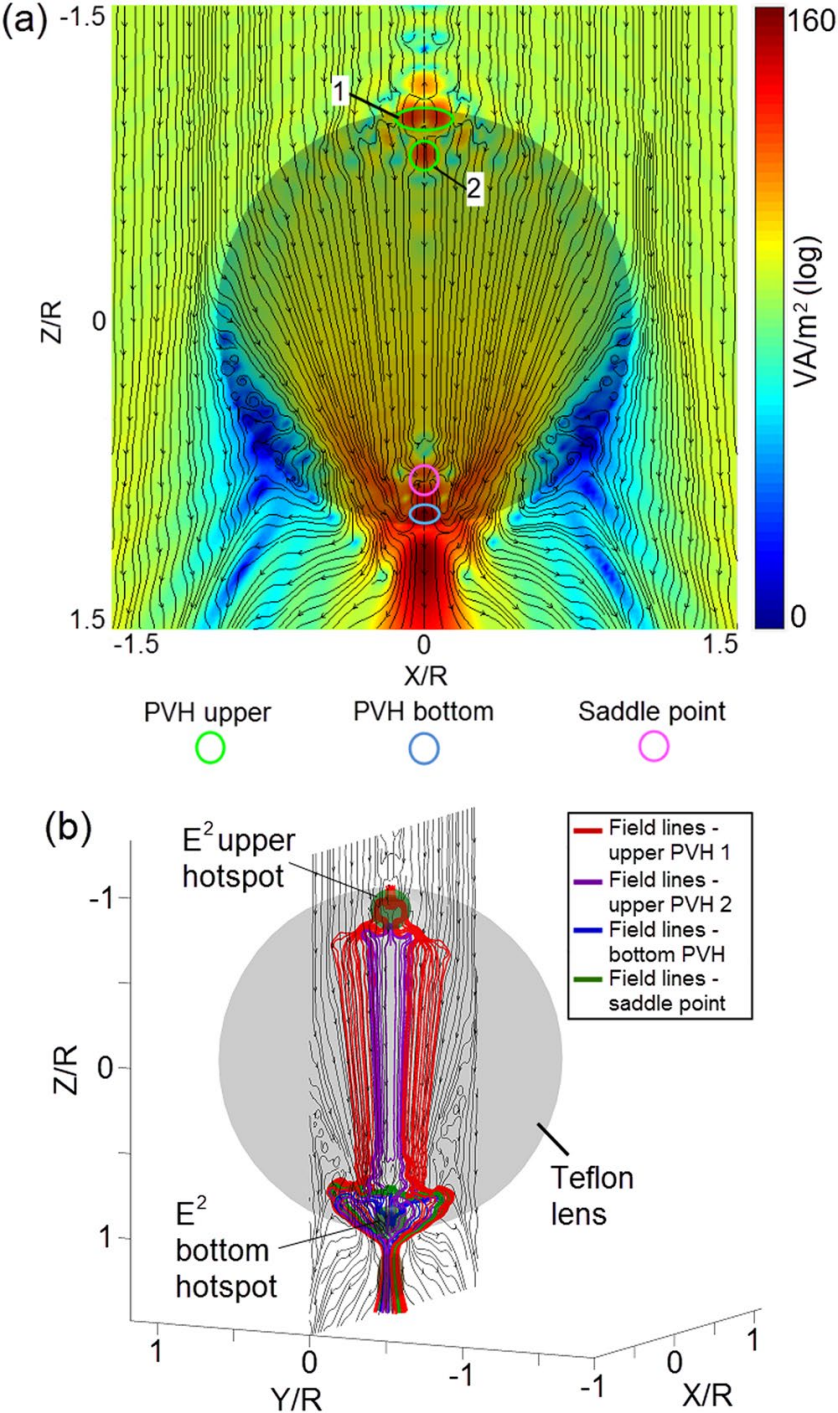
**(a)** The logarithmic 2D plot of Poynting vector of *xz* plane. **(b)** The 3D plot of Poynting vectors initiating at the critical points.

Since the layout of double polar hotspots in a sphere was overlooked in Lorenz-Mie theory and photonic jet effect so far, here we study the formation of these two hotspots. A logarithmic 2D plot of Poynting vector in the *xz* plane is shown in Fig. 2(a) for identification of the positions of the critical points (The distribution in the *xz* plane is less complex for analysis compared with that in the *yz* plane). We show that two Poynting vector hotspots (PVH) are marked as the green circles 1 and 2 near the upper pole, and a saddle point and a PVH are marked as the pink circle and the light blue circle near the bottom pole in Fig. 2(a), respectively. All these critical points are embraced by the polar hotspots of |*E|*^2^ field-intensity presented in Fig. 1(b)i. Also, the vortexes of Poynting vector appear at both flanks in the lower part of the sphere with the blue contours representing the small magnitude of Poynting vector. The largest magnitude of the Poynting vector is found in the jet area in Fig. 2(a), which is different from the |*E*|^2^ field-intensity shown in Fig. 1(b)i. The spatial tracking of Poynting vector field-lines takes place at the critical points – this is based on the information provided in Fig. 2(a), and the corresponding 3D plot is exhibited in Fig. 2(b).

We illustrate the Teflon sphere as a transparent grey circle shown in Fig. 2(b). The 2D plot of Poynting vector along the *xz* plane also crosses the sphere. The spaces of two polar hotspots of |*E|*^2^ field-intensity are illustrated as two small transparent green-colour spheres with the radius of 0.1*a* to indicate the positions of 3D field-line tracking in Fig. 2(b). The 3D field-lines originating from the different critical points are distinct by multiple colours, such as the red line for the PVH upper 1, the purple line for the PVH upper 2, the blue line for the PVH bottom, and the green line for the saddle point. Due to the high density of Poynting vector field-lines, we do not show the arrows on each of them by intention (Fig. 2(b)), but all directions of arrows are from top to down in this case. We note, that these field-lines form a large ‘heart-shape’ circulation in the sphere to relate two polar hotspots before convergence to a jet below the Teflon sphere. In addition, the field-lines of the PVH upper 1 (red-colour lines in Fig. 2(b)) shape into a small circulation around the upper pole which has a ‘bottleneck’ area at the position of the PVH upper 2.

**Figure 3:**
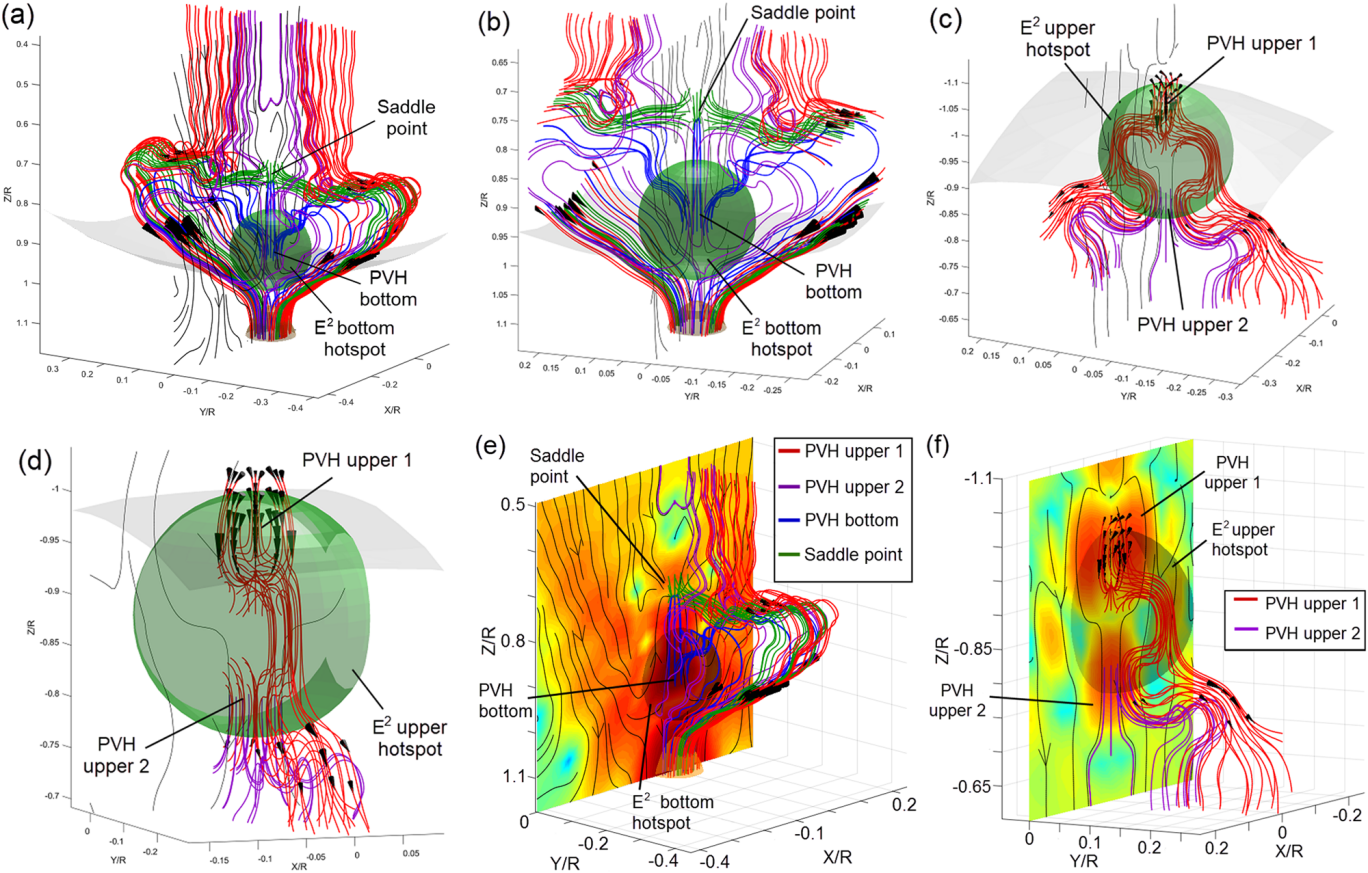
The zoomed maps of the field-lines for the bottom circulation **(a,b)** and the upper circulation **(c,d)** at the different angles and their relative positions to the critical points **(e,f)**.

The Fig. 3 shows the zoomed pictures of two circulations of the Poynting vector field-lines at different angles. The Fig. 3(a,b) show that the bottom hotspot of |*E|*^2^ field-intensity (the green-colour transparent sphere in Fig. 3(a,b)) is ‘wrapped’ inside the large ‘heart-shape’ circulation of Poynting vector field-lines, and its location coincides with that for the PVH bottom as all blue-colour field-lines are originally launched at the centre of the green-colour transparent sphere. It is interesting that the green-colour field-lines from the saddle point simultaneously grow to both directions against the *xz* plane, which means that the saddle point shown in Figs. 2 and 3 is an intersection of Poynting vector where the power flow consisting in the central plane escapes to the other locations in the Teflon sphere. The pattern of the upper small circulation is different from that of the large ‘heart-shape’ pattern. The PVH upper 1 and 2 are situated in the upper hotspot of *E*^2^ field-intensity, as shown in Fig. 3(c,d).

The field-lines around the PVH upper 1 are dense in terms of arrows, indicating significant direction changes of Poynting vectors in that area. These arrows configure three streams to whirl inward in the *xz* plane, as shown in Fig. 3(d), and then merge with the field-lines of PVH upper 2 (purple-colour) to form the circulation and flow down together, as shown in Fig. 3(c). The relative positions of 3D Poynting vector field-lines to the critical points and the PVHs are shown in Fig. 3(e,f) for the bottom and upper circulations, respectively. The multi-time lateral movement of the Poynting vector along the *y*-axis direction would cause the high magnitude for the PVHs at the saddle point and the centre of the bottom hotspot of *|E|*^2^ field-intensity, as shown in Fig. 3(e). However, the formation of the PVH upper 1 and 2 is due to the repeatedly longitudinal movement of Poynting vector at the positions of the whirl and bottleneck of the small circulation, as shown in Fig. 3(f). Therefore, two multi-time circulations of Poynting vector happen simultaneously in the sphere, and the heart-like pattern provides an enormous angular variation within an enormous circulation range. This could result in the giant |*E|*^2^ field-intensity enhancement at the poles for the specifically sized sphere, as shown in Fig. 1.

**Figure 4:**
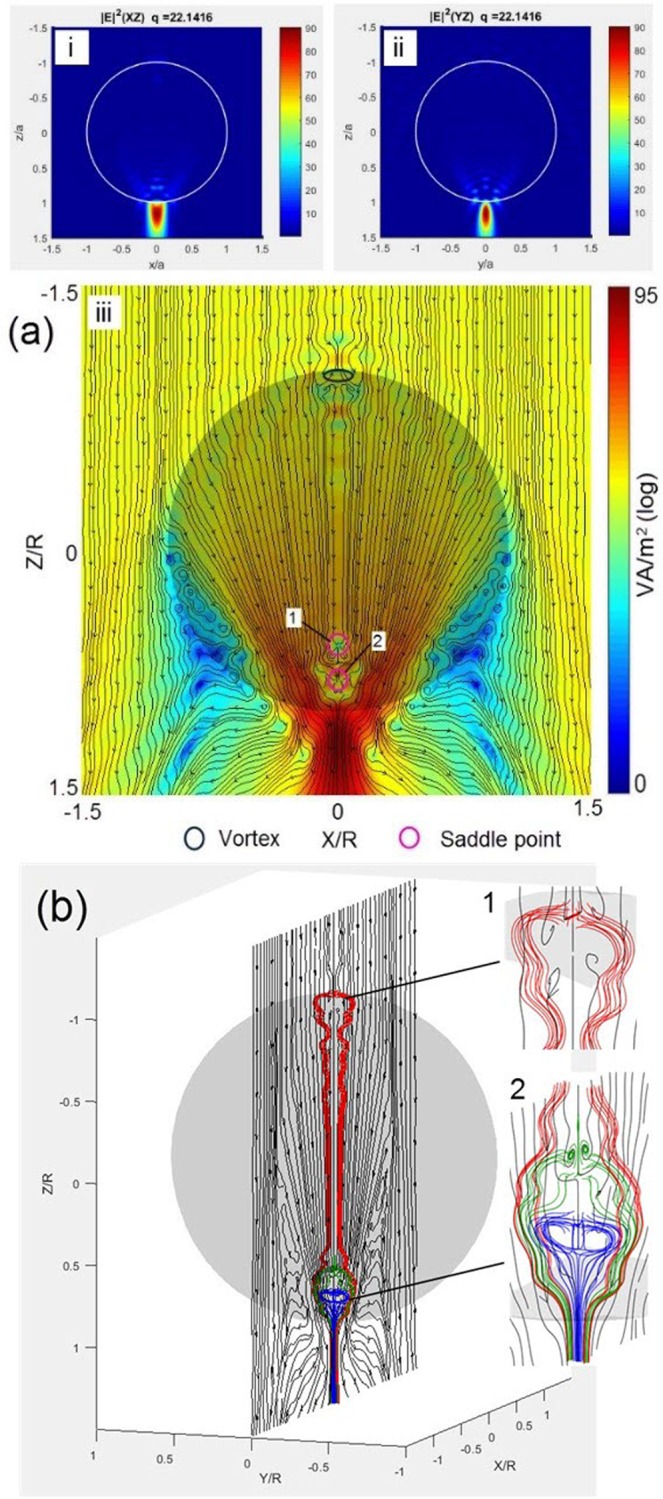
The distributions of |*E|*^2^ field-intensity and Poynting vector **(a)** and the 3D plot of Poynting vectors initiating at the critical points **(b)** for the sphere of *q* = 22.14159.

Meanwhile, it is noted that the aforementioned giant enhancement of |*E|*^2^ field-intensity and large ‘heart-shape’ circulation of the Poynting vector cannot be found in the Teflon spheres with the neighbouring size parameters. The distributions of |*E|*^2^ field-intensity and Poynting vectors for the sphere with the size parameter of *q* = 22.14159 are plotted as an example to demonstrate this contrast in Fig. 4(a,b), respectively. It is demonstrated that a typical photonic jet instead of two polar hotspots appears in the distributions of |*E|*^2^ field-intensity in the *xz* and *yz* planes for the sphere of *q* = 22.14159, as shown in Fig. 4(a)i,ii. Also, three critical points (one vortex and two saddle points) are indicated in Fig. 4(a)iii to perform 3D field-line tracking, however, there is only a single small circulation found in the lower part of the sphere, as shown in Fig. 4(b) and its insets. The corresponding angular variation and circulation range are much smaller compared to those for the sphere of *q* = 22.24159 with the giant field-intensity enhancement which are shown in Figs. 2 and 3. In addition, a model of high-index silicon sphere (*n* = 3.55 in the band of near-infrared^33^) was created using the same algorithm (the results are shown in Figs. S1 and S2 in the supplement of the paper). The similar field-intensity enhancement and large ‘heart-shape’ circulation of the Poynting vector are also found in the specifically sized spheres.

## Conclusion

To conclude, we proposed a 3D mapping technology innovatively used to track field-lines passing the critical points of Poynting vector distribution for investigation of a significant field-intensity enhancement existing around the poles of dielectric spheres. We discovered an astonishing large ‘heart-like’ multi-time circulation of Poynting vector 3D field-lines, which cannot be characterised by conventional 2D mapping analysis. Giant angular variation and circulation range are considered to be the primary causes of this phenomenon. Our findings are expected to deepen the knowledge in formation of fascinating effects such as photonic jet and hotspots. We stress that the corresponding giant field-intensity enhancement can be intentionally triggered in the multiple spectral ranges by tuning the wavelength of an incident plane wave illuminating the spherical particle under the specific parameters of refractive index and size. We anticipate that this effect and the technique of 3D Poynting vector track will contribute to the researches in field-enhancement phenomena, suppression of scattering, Fano resonance, and chiral material.

## Methods

In this study, we implement the complete Lorenz-Mie formulas in FORTRAN and MATLAB scripts to automatically sweep *q* values and study the evolution of peak |*E|*^2^ field-intensity enhancement within π and 20π (3.14159 to 62.8318) with a resolution of 0.1. A plane wave polarized in *x-*axis direction illuminates the Teflon sphere along the *z*-axis as shown in Fig. 1(a). The size parameter of the Lorenz-Mie theory, *q*, can be expressed as^34^,

1 $$q=\frac{2\pi a}{\lambda },$$

where *a* is Teflon sphere radius, *λ* is the wavelength of the incident plane wave. Due to a relatively constant relative permittivity, *ε*, of Teflon in the THz band (*ε* = *n* + *ki*), and simplify the calculation, values of *n* and *k* of Teflon are considered as constants in our algorithms. We consider the background medium as the air with index of *n* = 1. Here *n* of Teflon is defined as 1.43^32^, and *k* is 0 for a case of lossless sphere. In our algorithm, we spatially scan across the sphere centre from −5*a* to 5*a* along the incident direction of the plane wave to find the highest |*E|*^2^ field-intensity and its position in the sphere. Values of 500 points are collected per *a* in the sphere and its vicinity. Therefore, the total 5000 points are sampled per *q*. In addition, we plot the Poynting vector distribution along the cross-section of the sphere. All field-lines passing through the critical points in the high *|E|*^2^ field-intensity areas are automatically tracked by the algorithm. The *|E|*^2^ field-intensity enhancement in the sphere is induced by the scattered plane wave and composed of contributions from multiple resonant order modes. Its strength is quantified and determined by the absolute values of the complex scattering wave coefficients, ${B}_{l}^{e}$ for electric field and ${B}_{l}^{m}$ for magnetic field^35^. These coefficients are expressed in the form:

2 $${B}_{l}^{e}={i}^{l+1}\frac{2l+1}{l(l+1)}\frac{\hat{n}{\psi }_{l}^{\text{'}}(q){\psi }_{l}(\hat{n}q)-{\psi }_{l}(q){\psi }_{l}^{\text{'}}(\hat{n}q)}{\hat{n}{\zeta }_{l}^{{(l)}^{\text{'}}}(q){\psi }_{l}(\hat{n}q)-{\zeta }_{l}^{(l)}(q){\psi }_{l}^{\text{'}}(\hat{n}q)},$$

3 $${B}_{l}^{m}={i}^{l+1}\frac{2l+1}{l(l+1)}\frac{\hat{n}{\psi }_{l}(q){\psi }_{l}^{\text{'}}(\hat{n}q)-{\psi }_{l}^{\text{'}}(q){\psi }_{l}(\hat{n}q)}{\hat{n}{\zeta }_{l}^{(l)}(q){\psi }_{l}^{\text{'}}(\hat{n}q)-{\zeta }_{l}^{{(l)}^{\text{'}}}(q){\psi }_{l}(\hat{n}q)},$$

where *l* is the order of mode, $\hat{n}\,$ is the complex refractive index of the sphere relative to the surrounding medium, and ${\psi }_{l}(q)$ and ${\zeta }_{l}^{(l)}(q)$ are defined by^35^,

4 $${\psi }_{l}(q)=\sqrt{\frac{\pi q}{2}}\,{J}_{l+\frac{1}{2}}(q),$$

5 $${\zeta }_{l}^{(l)}(q)={\psi }_{l}(q)-i{\chi }_{l}(q)=\sqrt{\frac{\pi q}{2}}\,{H}_{l+\frac{1}{2}}^{(1)}(q),$$

6 $${\chi }_{l}(q)=-\sqrt{\frac{\pi q}{2}}{N}_{l+\frac{1}{2}}(q),$$

where ${J}_{l+\frac{1}{2}}(q)$, ${H}_{l+\frac{1}{2}}^{(1)}(q)$, and ${N}_{l+\frac{1}{2}}(q)$ are the Bessel functions, the Hankel functions, and the Neumann functions, respectively^35^. Multiple order modes of resonance are stimulated in the sphere at the same time, and their collective contribution on |*E|*^2^ field-intensity enhancement is shown in Fig. 1(a). Also, amplitude of individual mode would finally approach 0 at a certain order. An empirical formula identifying this nearly 0 contribution order of mode, *l*_0_, is given by^5^,

7 $${l}_{0}\approx q+4.3{q}^{\frac{1}{3}}+1,$$

The iterative calculation of collective enhancement is terminated when *l*_0_ is reached.

## Supplementary information

Supplementary materials.
