# Supplementary material for: Full three-dimensional Poynting vector flow analysis of great field-intensity enhancement in specifically sized spherical-particles
Source: Sci Rep. 2019 Dec 27;9:20224. doi: 10.1038/s41598-019-56761-9 (PMC6934590; doi:10.1038/s41598-019-56761-9)
Supplement: Supplementary file 1 — Supplementary materials. [file 41598_2019_56761_MOESM1_ESM.docx]

Full three-dimensional Poynting vector flow analysis of great field-intensity enhancement in specifically sized spherical-particles

Liyang Yue^1,^*, Bing Yan^1^, James N. Monks^1^, Rakesh Dhama^1^, Chunlei Jiang^2^, Oleg V. Minin^3,4^, Igor V. Minin^3,4,^*, and Zengbo Wang^1,^*

^1^School of Computer Science and Electronic Engineering, Bangor University, Dean Street, Bangor, Gwynedd, LL57 1UT, UK

^2^College of Electrical and Information Engineering, Northeast Petroleum University, Daqing, China

^3^National Research Tomsk Polytechnic University, Lenin Ave., 30, Tomsk, 634050, Russia ^4^National Research Tomsk State University, Lenin Ave., 36, Tomsk, 634050, Russia

Supplement materials


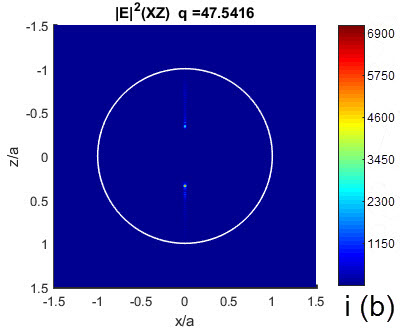

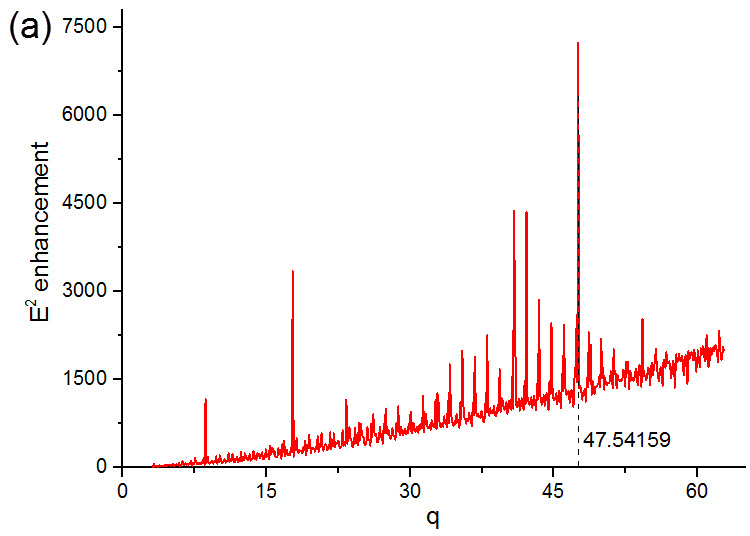


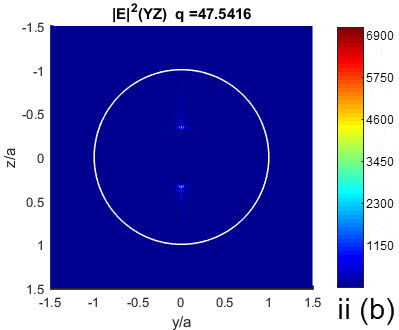


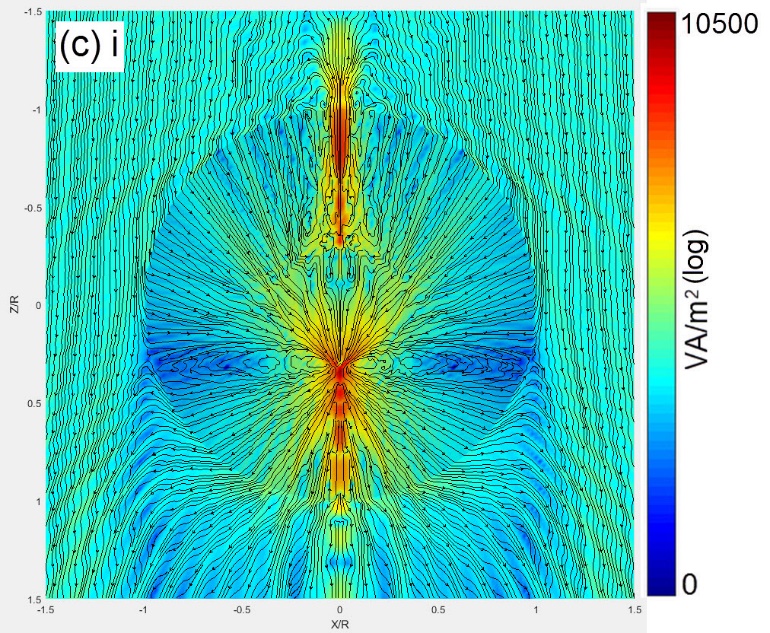


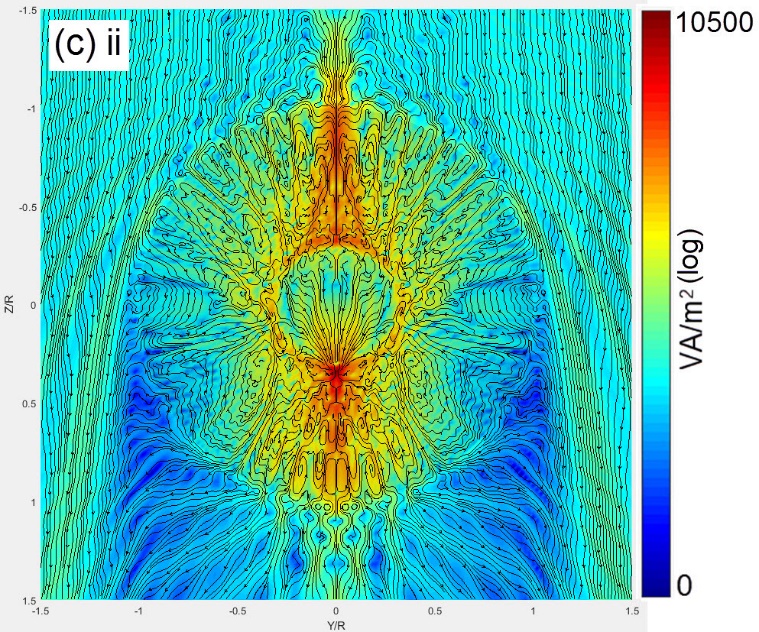


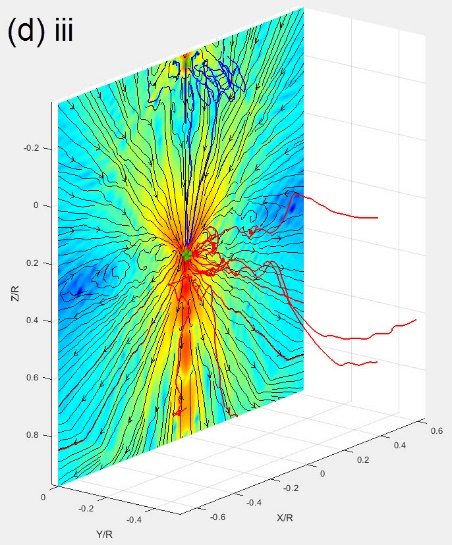

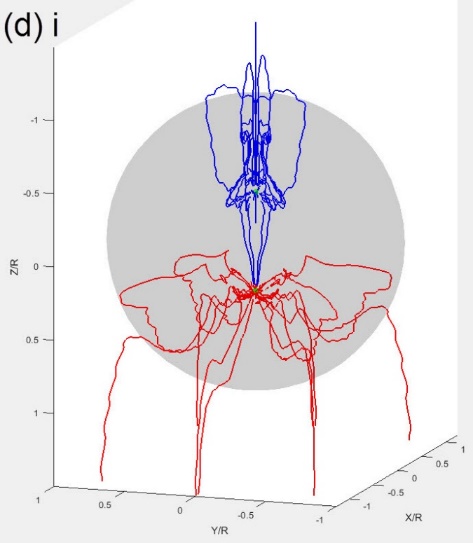

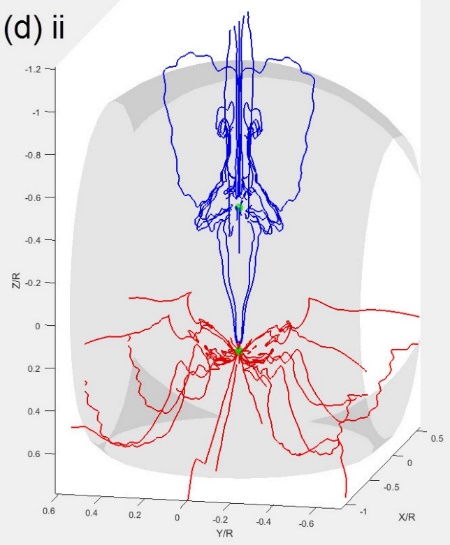


**Figure S1. (a)** Peak *|E|^2^* field-intensities for the silicon spheres (*n* = 3.55) versus size parameter, *q*. The distributions of |*E|^2^* field-intensity in the *xz* plane **(b) i** and the *yz* plane **(b) ii** when *q* = 47.54159. The logarithmic 2D plot of Poynting vector of *xz* plane **(c) i** and yz plane **(c) ii**. The 3D plot of Poynting vectors initiating at the critical points **(d) i**, **(d) ii**, and **d (iii)**.


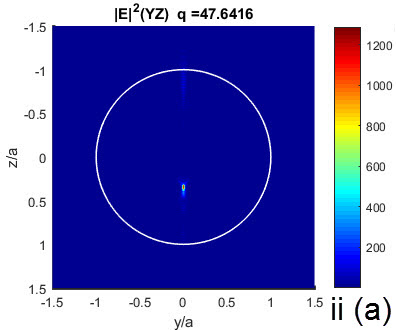

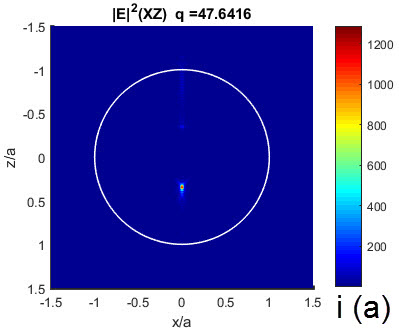


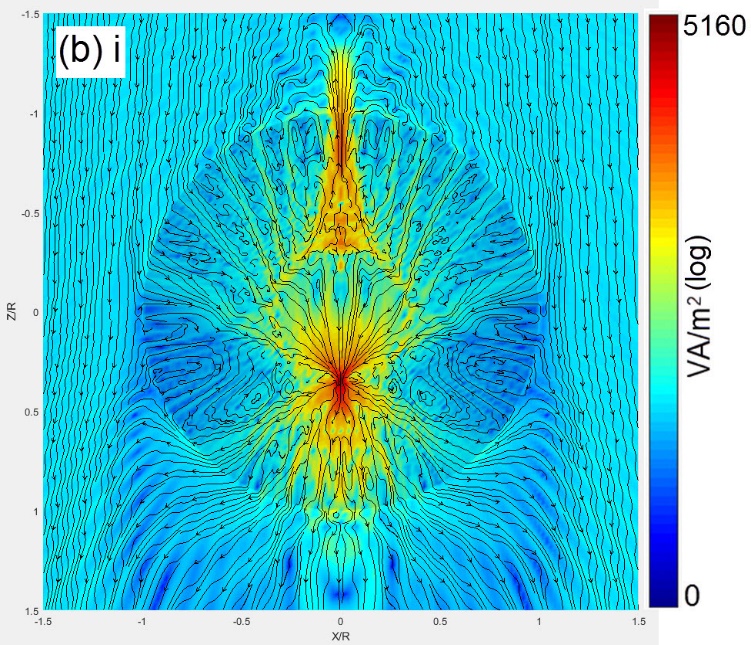


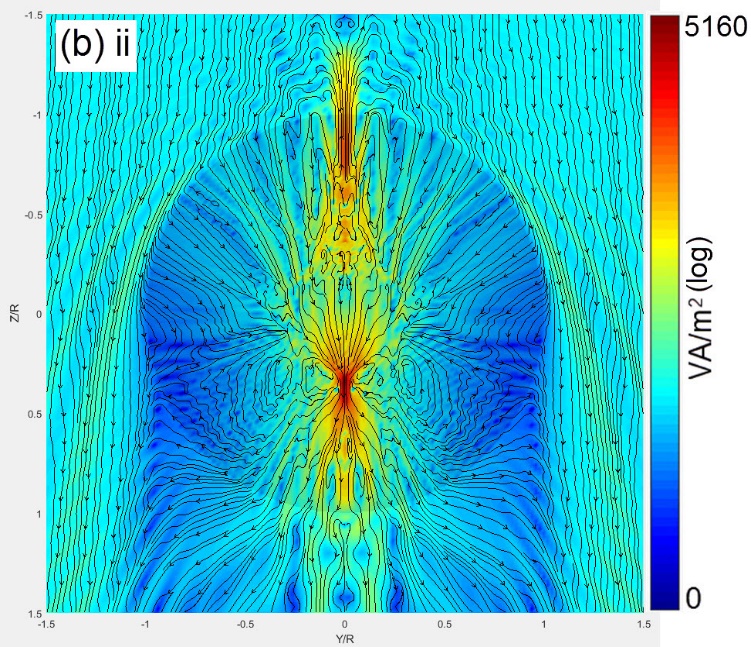


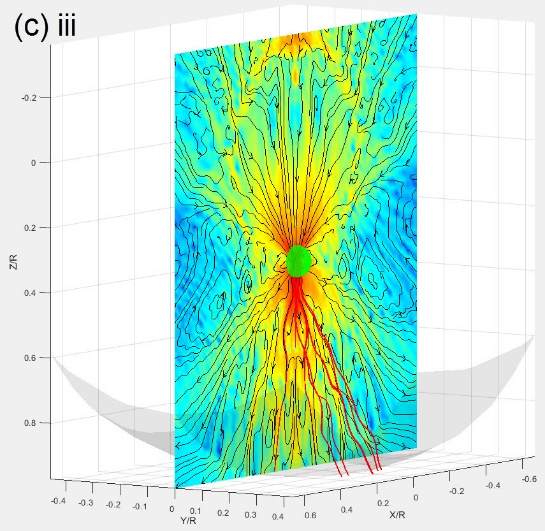

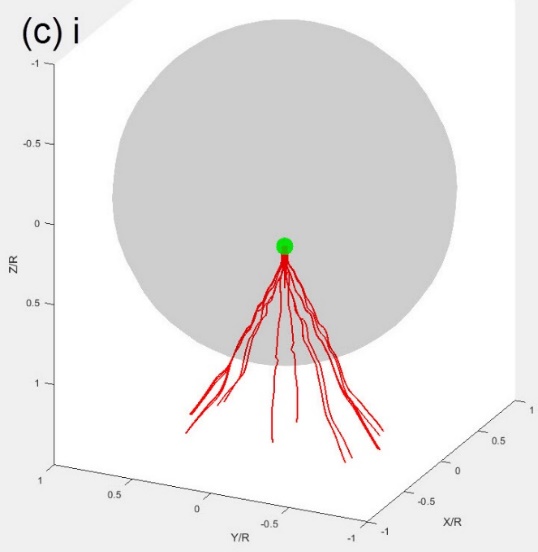

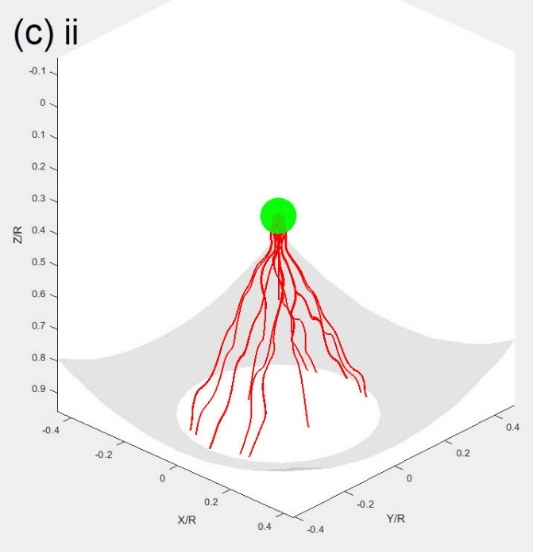


**Figure S2.** The distributions of |*E|^2^* field-intensity for the silicon sphere (*n* = 3.55) in the *xz* plane **(a) i** and the *yz* plane **(a) ii** when *q* = 47.64159. The logarithmic 2D plot of Poynting vector of *xz* plane **(b) i** and yz plane **(b) ii**. The 3D plot of Poynting vectors initiating at the critical points **(c) i**, **(c) ii**, and **c (iii)**.
